# Identification of *MYC* intron 2 regions that modulate expression

**DOI:** 10.1371/journal.pone.0296889

**Published:** 2024-01-18

**Authors:** Van S. Tompkins, Zheng Xue, Jake M. Peterson, Warren B. Rouse, Collin A. O’Leary, Walter N. Moss

**Affiliations:** Roy J. Carver Department of Biochemistry, Biophysics, and Molecular Biology, Iowa State University, Ames, Iowa, United States of America; BMSCE: BMS College of Engineering, INDIA

## Abstract

*MYC* pre-mRNA is spliced with high fidelity to produce the transcription factor known to regulate cellular differentiation, proliferation, apoptosis, and alternative splicing. The mechanisms underpinning the pre-mRNA splicing of *MYC*, however, remain mostly unexplored. In this study, we examined the interaction of heterogeneous nuclear ribonucleoprotein C (HNRNPC) with *MYC* intron 2. Building off published eCLIP studies, we confirmed this interaction with poly(U) regions in intron 2 of *MYC* and found that full binding is correlated with optimal protein production. The interaction appears to be compensatory, as mutational disruption of all three poly(U) regions was required to reduce both HNRNPC binding capacity and fidelity of either splicing or translation. Poly(U) sequences in *MYC* intron 2 were relatively conserved across sequences from several different species. Lastly, we identified a short sequence just upstream of an HNRNPC binding region that when removed enhances *MYC* translation.

## Introduction

We are in an exciting new era where “undruggable” proteins, such as the transcription factor MYC, have potential to be targeted at the mRNA level [1]. Initially identified as a cellular homologue to the oncogene of the avian myelocytomatosis virus (v-*myc)* [2], MYC is well-known, playing a central role in cellular expression, proliferation, differentiation, and apoptosis. Its elevated or deregulated expression has been associated with a wide range of aggressive human cancers including breast carcinoma, lung carcinoma, and neuroblastoma [3–6]; additionally, MYC expression is known to influence many of the hallmarks of cancer [7, 8]. Despite the numerous scientific publications dedicated to understanding the gene, no clinical MYC-specific inhibitor is yet available. Though the work of Tong et al. (2023) [1] is a promising lead toward this end, more investigation is needed to better understand *MYC*.

*MYC* RNA is subject to a wide variety of controls, ranging from differential transcriptional start sites to internal ribosomal entry sites (IRES) driving expression from alternative start codons [9, 10]. Our own previous studies identified several regions of *MYC* mRNA secondary structure that may regulate expression [11]. More recently, RNA methylation (m^6^A) was identified as a regulator of MYC expression [12]. Though evidence suggests that oncogenic activity of *MYC* is at least partially dependent on alternative splicing of other genes [13, 14], there is no evidence that *MYC* itself undergoes alternative splicing. The high splicing fidelity observed in *MYC* may be partially attributed to the simplicity of its gene architecture, consisting of three exons and two introns; however, the mechanisms underlying the faithful splicing and expression of MYC largely remain a mystery.

**Fig 1:**
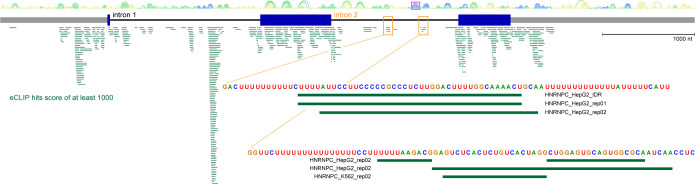
Identifying HNRNPC binding to *MYC* intron 2 in eCLIP data using IGV-ScanFold. *MYC* pre-mRNA is represented by gray boxes (5′ and 3′ UTRs), blue boxes (CDS), and black lines (introns). Above are the base-pairing results from IGV-ScanFold, where base pairs are indicated with arcs and colors represent average z-scores as follows: yellow is 0 to >-1, green is -1 to >-2, blue is <-2. Green boxes represent eCLIP hits across *MYC* pre-mRNA with a score of 1000 or greater for a variety of RNA binding proteins from different cells lines. The orange boxes denote two regions in intron 2 where HNRNPC binding was observed. The sequences identified within those regions are magnified below. The magenta box indicates a ScanFold-predicted hairpin that is discussed below.

Many RNA binding proteins (RBPs) for *MYC* pre-mRNA have been identified. Enhanced cross-linking immunoprecipitation (eCLIP) reported by Van Nostrand et al. (2020) evaluated over 70 RBPs across multiple cell lines [15]. Using a tool our lab developed, IGV-ScanFold [16], we visualized the most robust eCLIP results and observed a paucity of RBPs binding intron 2 versus intron 1 (Fig 1). Of the few high-scoring RBPs in intron 2, the heterogeneous nuclear ribonucleoprotein C (HNRNPC1/C2, collectively referred to hereafter as HNRNPC) stood out because it bound multiple regions in intron 2 (Fig 1). Furthermore, HNRNPC plays a role in RNA splicing [17] and has been shown to bind *MYC* mRNA and regulate its expression [18]. HNRNPC preferentially binds poly(U) regions of transcripts, where the transcript binding location may determine how it functionally regulates that RNA [19]. It also has been shown to bind near and be regulated by m^6^A sites [20]. Non-splicing related functions for HNRNPC have also been described, such as the binding of HNRNPC to the mature *MYC* transcript between two alternative start codons–the primary CUG in the first exon and the AUG of the second exon–which can regulate IRES-dependent *MYC* expression in a cell cycle dependent manner [18].

This study verifies the binding of HNRNPC to *MYC* intron 2 in a manner dependent upon poly(U) regions. Our data suggests that this binding positively influences *MYC* splicing and expression, and that it is necessary to have multiple poly(U) regions in intron 2 for this. Further, we identify a six-nucleotide sequence near one of the HNRNPC binding regions that negatively regulates *MYC* intron 2 splicing or expression.

## Materials and methods

### Cell culture

HeLa and MCF7 cells were incubated at 37°C and 5% CO_2_ and maintained in DMEM supplemented with 10% FBS, penicillin/streptomycin, and L-glutamine (Gibco, Thermo Fisher). Cells were passaged at 60–100% confluence and used between passages 5–15. MCF7 cells were purchased from ATCC, whereas HeLa cells were obtained from the Iowa State Hybridoma Facility (Ames, Iowa). Both cell lines were confirmed to be mycoplasma free by PCR testing [21].

### RNA Immunoprecipitation (RIP)

HeLa cells were treated for 2 h with 1 μM pladienolide B (PladB) to inhibit splicing prior to formaldehyde (0.25%) cross-linking (in DPBS) for 10 min at room temperature and quenching with 5 mL of 125 mM glycine in DPBS. Cells were then rinsed with 5 mL of cold DPBS twice before lysis using 500 μL of RIPA lysis buffer (50 mM TrisHCl; pH 8.0; 1 mM EDTA; 150 mM NaCl; 100 μM Na_3_VO_4_; 1.0% NP-40; 1.0% sodium deoxycholate; 0.1% SDS) containing protease/RNase inhibitors (1:100 HALT; 1 mM PMSF; 300 U/mL RNaseOUT) (ThermoFisher) per two 100 mm dishes. A cell lifter was used to transfer the lysing cells to 1.5 mL microfuge tubes on ice. Lysates were sonicated on ice once at 50% amplitude for 5 sec prior to centrifugation to clear the lysate (15,000xg, 10 min, 4°C). Supernatant was diluted 2.5-fold with detergent-free lysis buffer containing 300 U/mL of RNaseOUT, followed by pre-clearing with protein A/G-PLUS agarose beads (100 μL) for 1 h rotating at 4°C. 500 μL lysate was incubated with each of anti-hnRNPC1/2 (sc32308, Santa Cruz Biotechnology) or isotype-matched mouse IgG antibody for 1 h at 4°C before the addition of A/G- PLUS agarose beads (25 μL) for an additional hour at 4°C on rotisserie. For input samples, 40% of the volume added for each antibody of the pre-cleared lysate was set aside on ice for RNA isolation. Beads were washed four times with cold DPBS (1 mL each) before TRIzol was added (to the input as well). Samples were then incubated for 5 min at room temperature prior to storage at -80°C or used directly in RNA isolation.

### RNA isolation, RT-PCR & qPCR

**Fig 2:**
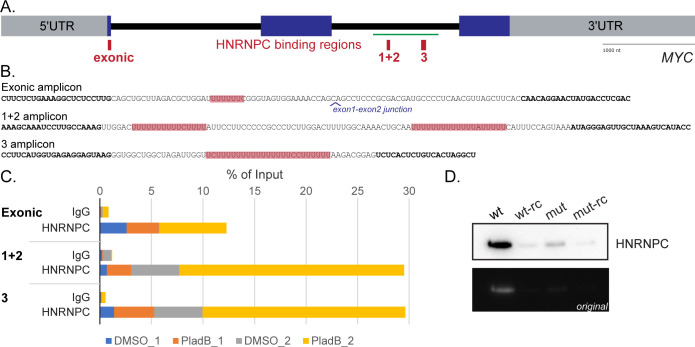
HNRNPC binding to *MYC* intron 2. Schematic of *MYC* pre-mRNA **(A)** and RIP amplicon sequences **(B)** that note, in red, the demonstrated exonic binding region of HNRNPC and the eCLIP-associated poly(U) regions. PCR primer binding is denoted by the bold font. **C)** The results of two independent HeLa cell RIP experiments, each with (PladB) and without (DMSO) 1 μM pladienolide B treatment for 2h, are graphed as a percentage of input after qPCR for the indicated regions. **D)** Immunoblot for HNRNPC following RNA-pulldown using MCF7 nuclear lysates and in vitro transcribed RNA (green bar in (A)) of wild-type (wt) or mutated (mut; no more than three U residues in a row for all poly(U) regions in (B)). “RC” indicates the transcribed reverse-complement, and the original Fotodyne-acquired image is shown below the enhanced (for clarity only) inverted image above. Please see S1 Raw images for full blot images. This blot was performed once but see Fig 3B for verification of findings.

Prior to isolation, RIP TRIzol samples were heated to 65°C to reverse cross-linking. RNA isolations were then done for total RNA using TRIzol, QuantBio Phase-Lock Gel tubes, and a Zymo RNA Clean & Concentrate kit, per manufacturer’s instructions. Input lysate was eluted in a higher volume to make the input 10% at equal volumes to the immunoprecipitation (IP) samples for reverse transcription (RT). Controls for contaminating DNA (noRT) were done using the input lysate. Total RNA, random hexamers (IDT), and Superscript III (ThermoFisher) were used for RT. Relative abundance of transcripts across samples were measured by qPCR, using PowerUp SYBR Green Master Mix (ThermoFisher) on an Applied Biosystems QuantStudio 3 instrument (ThermoFisher) with the following primers (Fig 2; 5′ to 3′): HNRNPC 1+2 amplicon with AAAGCAAATCCTTGCCAAAGT (F) & GGTATGACTTTAGCAACTCCCTAT (R); HNRNPC 3 amplicon with CCTTCATGGTGAGAGGAGTAAG (F) & AGCCTAGTGACAGAGTGAGA (R); exonic amplicon with CTTCTCTGAAAGGCTCTCCTTG (F) & GTCGAGGTCATAGTTCCTGTTG (R). All oligonucleotides used in this study were purchased through Integrated DNA Technologies (IDT).

**Fig 3:**
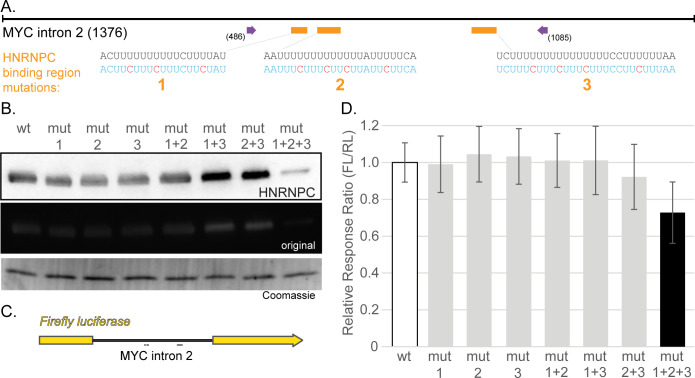
Poly(U) mutations in *MYC* intron 2 reduce HNRNPC binding and inhibit splicing. **A)** *MYC* Intron 2 is represented with attention to three poly(U) regions (orange boxes and sequences below) that were mutated. Biotinylated, *in vitro* transcribed RNA (region between purple arrows) was incubated with HeLa lysates followed by immunoblotting **(B)** for HNRNPC. Coomassie stain represents a type of loading control. The blot is representative of two independent experiments; both the uncropped original images and the result from the other independent experiment can be found in S1 Raw images. Mutation (mut) numbering indicates which regions shown in (A) were mutated for each. **C)** Schematic of the intron insertion reporter used for experiments. **D)** Results of dual luciferase assays, plotted as the ratio of Firefly (FL) to Renilla (RL) relative light units with mutation numbering as before. Data are from at least 12 independent transfections in HeLa cells performed on at least two separate days. Black bar indicates a significant difference from wild-type (wt; p < 0.05, two-tailed Student’s t-test).

### Cloning MYC intron 2 into pmirGLOi

Intron 2 of *MYC* was cloned as an intron into the firefly gene of a modified pmirGLO (Promega) reporter plasmid [22]. This was done using genomic DNA from HeLa cells isolated using Zymo’s Quick-DNA Miniprep kit. All subsequent PCR reactions used Q5 DNA polymerase (New England Biolabs, NEB). Intron 2 was PCR amplified (all primers 5′ to 3′) using oligonucleotides AGCAGCGACTCTGGTAAGCGAAG (F) & CCACAGAAACAACATCGATTTCTTCCTCATCTTC (R). To remove exon sequences and add overlap regions for HiFi reactions (NEB) primer extension and overlap PCR were employed. Extension reactions used primers AACAGTAGTGGCAGTACCGGATTGCCCAAGGTAAGCGAAGCCCGC (F) & ACAAGCGGTGCGGTGCGGTAGGGCTACGCCCTTTAAGAAAGGAAATAGAAATCACTCCT (R). Overlap included 5′ and 3′ linker oligos in the PCR reaction, CAAGACCGACTACCAGGGCTTCCAAAGCATGTACACCTTCGTGACTTCCCATTTGCCACCCGGCTTCAACGAGTACGACTTCGTGCCCGAGAGCTTCGACCGGGACAAAACCATCGCCCTGATCATGAACAGTAGTGGCAGTACCGGATTGCCCAAG (5′ linker) & TGCCGAAGCCGTGGTGAAATGGCACCACGCTGAGGATAGCGGTGTCGGGGATGATCTGGTTGCCGAAGATGGGGTCGCGGGCATGACTGAATCGGACACAAGCGGTGCGGTGCGGTAGGGCTACGCC (3′ linker), respectively, with end primers CCGACTACCAGGGCTTCCAAAGCAT (F) & CCGAAGCCGTGGTGAAAT (R). HiFi reactions used BsrGI and BbvCI (NEB) digested pmirGLOi [22] with the extended *MYC* intron 2 and were transformed into NEB-alpha cells. Colonies were PCR screened prior to miniprep (Qiaprep, Qiagen) and sequence verification (Iowa State University DNA Facility).

### Cloning of targeted mutations into reporter plasmid

We generated all mutant constructs of *MYC* intron 2 using two-fragment overlap PCR with primers containing the mutations prior to full intron HiFi cloning. For brevity, only the forward mutated primers (5′ to 3′) are shown here: mutant HNRNPC region 1 GTTGGACTTCTTTCTTTCTTCTATTCCTTCCC; mutant HNRNPC region 2 CAAAACTGCAATTTCTTTCTTCTTATTCTTCATTTCCAGTAAAATAG; mutant HNRNPC region 3 GGCTAGATTGGTTCTTTCTTTCTTTCTTTCCTTCTTTAAGACGGAGTCTC. The final product was made with the same end primers as for cloning the wild-type intron.

### Preparation of nuclear (MCF7) and whole-cell (HeLa) extracts

Cells were trypsinized, counted, centrifuged for 3 min at 200xg, washed once with ice cold DPBS, and pelleted again. Pellets were resuspended in ice cold hypotonic lysis buffer (HLB: 10 mM Tris, pH 7.5; 1.5 mM MgCl_2_; 10 mM KCl) for cellular fractionation or polysome extraction buffer (PEB: 20 mM Tris, pH 7.5; 100 mM KCl; 5 mM MgCl_2_; 0.5% NP-40 [23]) for whole-cell extract at 10^7^ c/mL and each containing 1 mM PMSF, HALT, 200 U/mL RNaseOUT. Each was incubated on ice for 15 min prior to Dounce homogenization (methanol-cleaned glass). For the whole-cell lysate, homogenate was spun at 15,000xg for 10 min at 4°C prior to collection of the supernatant. For fractionation, homogenate was spun for 10 min at 1,000xg and 4°C, supernatants were collected as the cytoplasmic fraction, and the pellets were resuspended in ice cold PEB. These were homogenized again before spinning at 15,000xg for 10 min at 4°C for collection of the supernatant nuclear fraction. Using the BCA assay (ThermoFisher) concentrations of lysates were determined.

### *In vitro* transcription and RNA precipitation

Reporter plasmids with either wild-type (WT) or mutant (MUT) *MYC* intron 2 were used as PCR templates to make T7-containing DNA fragments encompassing all three eCLIP-recognized HNRNPC binding regions (Fig 3). T7 recognition sequences were incorporated into the forward or the reverse complement (negative control) strands for the WT or the reverse complement control, respectively: TAATACGACTCACTATAGGGCCAGTGAACTGCCTCAA (F) & CACCTGTAGTCCCAGCTACTT (R); GCCAGTGAACTGCCTCAA (F) & TAATACGACTCACTATAGGGCACCTGTAGTCCCAGCTACTT (R). PCR products were used for *in vitro* transcription with T7 RNA polymerase using a 1:4 ratio of biotin-14-CTP to CTP (ThermoFisher) to generate biotinylated WT and MUT *MYC* intron 2 RNA fragments. RNA was allowed to fold through slow cooling after heating to 70°C and 500 ng was used to verify the correct RNA product size via urea-PAGE (National Diagnostics Urea-Gel SequaGel) and SYBR Green II RNA Gel Stain (S1 Fig) (ThermoFisher). Two μg of each RNA was incubated with 2.0 mg of nuclear or whole-cell lysate for 30 min at room temperature in Tris EDTA NaCl Triton buffer (TENT; 10 mM Tris pH 8.0, 1 mM EDTA pH 8.0, 250 mM NaCl 0.5% Triton X-100) [23] prior to the addition of 30 μL of buffer-equilibrated streptavidin agarose bead slurry for an additional 30 min at room temperature. Beads were then washed four times with 1 mL of ice cold TENT buffer and spun at 7,000xg for 30 sec at 4°C. 40 μL of 1X Laemmli buffer with 2-mercaptoethanol was added to the beads for immunoblotting.

### Immunoblotting

SDS-PAGE gel electrophoresis was performed with a 4–15% gradient gel (BioRad) after samples were boiled for 5 min. Samples were transferred onto a PVDF membrane using Tobin buffer. 5% non-fat milk in Tris-buffered saline with Tween20 (TBST; 10 mM Tris pH 8.0, 150 mM NaCl 0.05% Tween20) was used for blocking (1 h room temperature) and antibody incubations. Primary antibody incubation occurred overnight at 4°C with anti-hnRNPC1/2 (1:200; sc32308; Santa Cruz Biotechnology) and gentle rocking (~60 rpm for all incubations). After overnight incubation, the membrane was washed four times with TBST for 5 min each, incubated with the secondary antibody (1:2000; Invitrogen; 62–6520) for 40 min, washed another four times with TBST, and washed once with TBS all for 5 min at room temperature and gently rocking. Pierce ECL western blotting substrate (ThermoFisher) was applied for visualization, which was accomplished using a Fotodyne gel imager (2x2 binning, 150 gain, ~30-minute exposure times).

### Dual luciferase assay

*MYC* intron 2 (WT or MUT) containing pmirGLOi vectors were transfected (Lipofectamine 3000; Invitrogen) into HeLa cells at equivalent levels (5 ng/well) into each of 6 wells of a 96-well dish. Cells were plated 24 h prior at 20,000 c/well. Twenty-four hours post-transfection, cells were fed with 100 μL of DMEM and incubated another 24 h. Promega’s Dual Luciferase Reagent Assay kit was used per manufacturer’s protocol and read on a Promega GloMax Discovery instrument. The ratio of firefly luciferase to Renilla luciferase relative light units (RLUs), or relative response ratios (RRR), was calculated for each well.

Statistics were performed on the results of the dual luciferase assay based on comparison of the mean values using an unpaired Student’s t-test. Normality and equal variance were assumed, whereas directionality was not (two-tailed). Comparisons exhibiting a p-value < 0.05 were considered significant. Microsoft Excel was used for calculations.

### MYC orthologue intron 2 comparisons

Using Ensembl, intron sequences from various species were downloaded after a “MYC” search of representative sequences based on sequence and source variety. Only sequences that were from second introns with at least a two-intron genomic architecture were included, except for human *MYCL* (36 orthologues, 2 paralogues). All 39 sequences (including *MYC*) were aligned using MAFFT [24] and a cladogram was generated using Archaeopteryx [25]. Poly(U) regions were extracted using a Bing AI-generated python script, and distance calculations and graphs were done in Microsoft Excel (Microsoft).

Intron sequences were analyzed using ScanFold 2.0 [16] with default settings (no probing data, 120 nucleotide windows, 1 nucleotide step-size, global refold on, extract -2 z-score structures). Data were downloaded and z-average -1 dot-bracket structures were visually compared using VARNA [26]. ScanFold output data are available in S1 File.

### 3D Modeling

Structural motifs with covariance and/or experimental results were modeled using SimRNA [27] (1,000 intervals of 16,000 steps each, 8 iterations of 10 replicas each, recording the lowest energy frames in each interval), with models clustered around 5 Å, 7 Å, and 10 Å RMSD, as well as the sequence length divided by 10 (the latter technique recommended by Boniecki et al., 2016) [27]. The five largest clusters for each RMSD (with at least 5% of the total structures) were then converted back to all-atom models and refined using Quick Refinement of Nucleic Acid Structures (QRNAS) [28]. Refined models were then visualized and aligned using ChimeraX [29, 30].

## Results

### HNRNPC interacts with MYC intron 2 through poly(U) regions

To validate the eCLIP data for HNRNPC binding to intron 2 (Fig 1) of *MYC* pre-mRNA (Fig 2A) [15], RNA immunoprecipitation (RIP) from HeLa cells was performed. We designed and validated qPCR primers over the regions of interest (Fig 2B), including a previously reported exonic binding region as a positive control [18]. We designed and attempted primer validation of primers between intron 2 HNRNPC binding regions 1 and 2, but the primer efficiencies were not adequate, so amplification was done over both regions in one reaction as indicated. Indeed, we observed enrichment of exon 1 mRNA after immunoprecipitation with an antibody against HNRNPC, as well as increased association with both tested intron 2 pre-mRNA regions (Fig 2C). Such enrichment was also observed in cells treated with PladB, a splicing inhibitor that targets SF3B1 and allows for pre-mRNA intronic retention [31].

MCF7 nuclear lysates were used in precipitations with biotinylated RNA followed by immunoblotting to confirm the association (Fig 2D). Biotin-CTP (1:4 with CTP) was used in T7-driven *in vitro* transcription to generate a portion of *MYC* intron 2 RNA that encompassed all three binding regions (green bar in Fig 2A; between purple arrows in Fig 3A). As controls, reverse complement RNA was used. RNA containing mutations in all three binding regions (no more than 3 consecutive U residues; see Fig 3A) showed severely diminished binding compared to WT after immunoblotting with an antibody specific for HNRNPC.

### HNRNPC binds through different intron 2 poly(U) regions to affect MYC expression

Whether any of the *MYC* intron 2 binding regions preferentially bound HNRNPC was then examined. To do so, *in vitro* transcribed, biotinylated RNAs containing mutations in the different regions, either singularly or in all possible combinations, were generated. These were incubated with whole cell lysates from HeLa cells followed by precipitation and immunoblotting for HNRNPC (Fig 3A and 3B). Only the simultaneous mutation of all three binding regions diminished binding to HNRNPC (Fig 3B).

We also asked whether the binding of HNRNPC to intron 2 of *MYC* could alter splicing of *MYC* pre-mRNA. To this end, a dual luciferase plasmid was generated containing *MYC* intron 2 inserted into the Firefly coding region (Fig 3C) at a location previously demonstrated not to adversely affect the resultant protein [22]. This approach also allowed introduction of mutations to each of the binding regions. In HeLa cells, the dual luciferase assays indicated that any one of the binding regions alone was sufficient for optimal expression, but when all three HNRNPC binding regions were mutated, expression of the reporter and, presumably, the splicing efficiency was reduced (Fig 3D).

### Poly(U) regions are not uncommon in MYC intron 2

**Fig 4:**
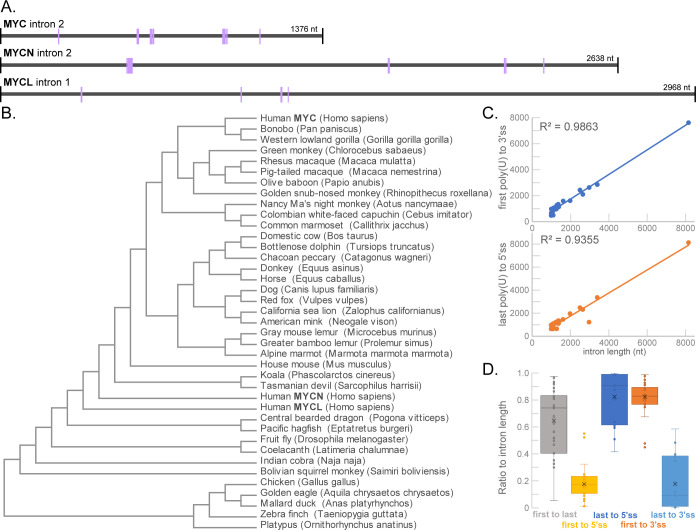
Poly(U) regions identified in other *MYC* introns. **A)** Comparison of introns for paralogues of human *MYC* showing poly(U) regions (purple) with at least five consecutive U residues. **B)** Cladogram after MAFFT alignment of *MYC* intron 2 sequences (exception of human *MYCL* that only has one intron) from various species. **C)** Plots of the length of the intron versus either (top) the distance of the first poly(U) to the 3′ splice site (3′ss) or (bottom) the distance of the last poly(U) to the 5′ splice site (5′ss) demonstrate strong positive linear relationships. **D)** Box plot demonstrating the consistently high ratios between intron length and distances of indicated poly(U) regions to the 5′ss and the 3′ss of *MYC* across the species examined. Values represented in graphs are found in S3 File.

Trying to get a better idea of the extent of poly(U) regions that HNRNPC could potentially bind in *MYC* introns, the introns of the two other human paralogues, *MYCL* and *MYCN*, were examined. *MYCN* has a two-intron architecture like *MYC*, but *MYCL* contains only a single intron. Despite this architectural difference, there are four independent locations of 5 or more consecutive U residues (poly(5U)) to which HNRNPC could potentially bind in the single *MYCL* intron (Fig 4A). *MYC* contains seven of these regions with five of them falling within the three HNRNPC binding regions we explored above–the other two are farther up- and downstream, respectively, and remain unexplored. *MYCN* intron 2 also contained seven poly(5U) regions. Each intron contained at least two stretches of seven or more consecutive uridine residues.

The fact that all three contained several poly(5U) segments made us ask whether the existence of these poly(U) regions was a common, conserved feature to *MYC* introns. Restricting the search to forms of *MYC* that contained a second intron–except for the inclusion of human *MYCL*–we found all poly(5U) regions and mapped their locations within the intron (S2 File). Within the 39 intronic sequences, 276 poly(5U) regions were identified with a minimum number of two per intron (mean = 7.1; median = 6). Indeed, a MAFFT alignment of these intronic sequences–representing a range of organisms (Fig 4B)–showed that a 65% threshold consensus sequence also contained two poly(U) regions (S2 Fig).

Simply considering the position of the first poly(U) region and the last poly(U) region for each intron, trends were observed. As expected, there was a strong positive correlation between the number of poly(U) regions and length of the intron (S3 File). Even stronger correlation coefficients were observed between length and distance from the last poly(U) region to the 5′ splice site (5′ss), distance from the first poly(U) region to the 3′ splice site (3′ss), and the distance interval between the first and last poly(U) regions, respectively (S3 File and Fig 4C). In line with this, ratios between intron length and distances of both the last poly(U) to the 5′ss and the first poly(U) to the 3′ss were consistently high (Fig 4D). These consistencies suggest an importance for the position of these poly(U) sequences relative to the splice sites.

### Short sequence just upstream of HNRNPC binding negatively affects MYC expression

**Fig 5:**
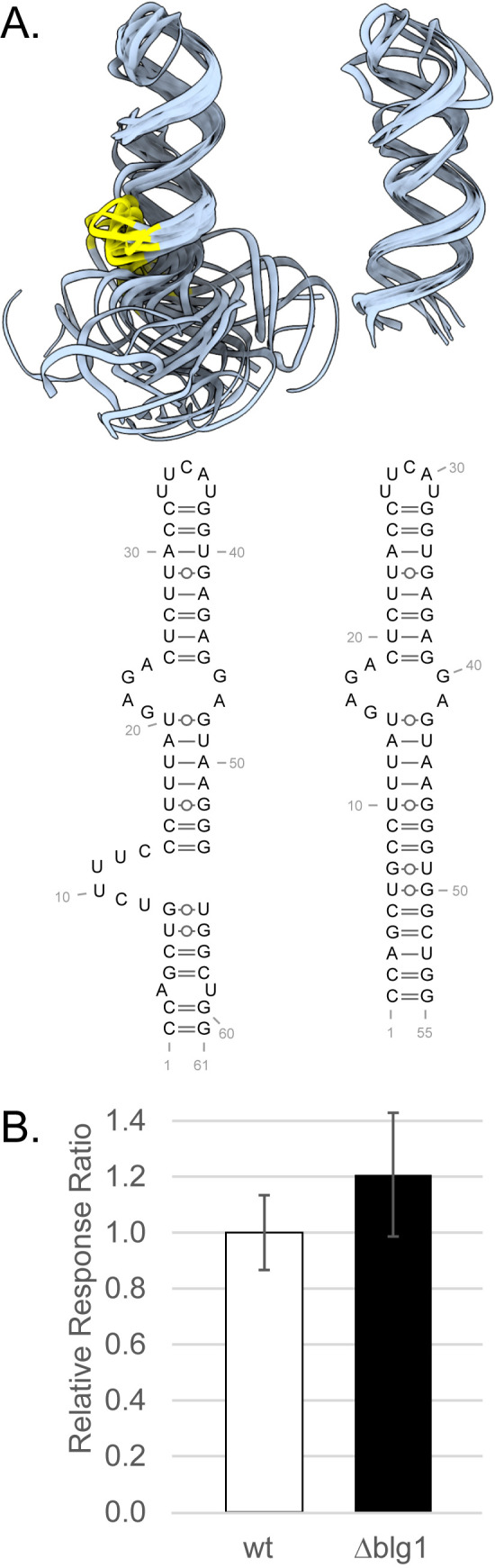
Modeled six-nucleotide bulge in a hairpin just upstream of HNRNPC binding region attenuates splicing. **A)** 3D modeling images of all > 5% modeled structures overlayed for each of the wild-type (left) and the deletion of bulge 1 (right) models. Yellow regions denote the backbone of the six nucleotides. The respective 2D model, determined by ScanFold, is shown below. **B)** Results from splicing reporter assays comparing MYC intron 2 wild-type (wt) and deletion of bulge 1 (Δblg1). Data represent average relative response ratio (firefly:renilla) from 12 independent transfections done at two independent times in HeLa cells. Data are significant at p < 0.05 by Student’s t-test (2-tailed).

Several of these comparative species were primates (16/39 including 3 human sequences). Using ScanFold 2.0 [16], secondary structural models of sequences around the poly(U) HNRNPC binding regions were generated for these primates and compared to identify conserved elements with sequence-ordered thermodynamic stability (S1 File). Old world primates (including human) were found to have an RNA hairpin with consistent asymmetrical bulges just upstream of an HNRNPC binding region (S3 Fig). Based on this and knowing that elements binding near HNRNPC can be important for the regulatory functions of HNRNPC or the binding elements [20], the human hairpin was modeled in three dimensions using coarse-grained cluster models from SimRNA [27] and the all-atom representations were refined using QRNAS [28]. We qualitatively aligned the best representative models (as defined in the Methods) and observed a highly flexible region predominantly caused by a one-sided asymmetrical bulge (Fig 5A, left; S4 File). Remodeling the hairpin when the six-nucleotide bulge was removed greatly reduced variety and flexibility in the cluster models compared to the wild-type models (Fig 5A, right; compare S4 and S5 Files). Using our reporter assay, this region was mutated to remove the six nucleotides of the bulge. A slight but significant increase in the relative response ratio (Fig 5B) was observed, indicating that the nucleotides that make up this predicted bulge region exert an inhibitory effect on *MYC* splicing or translation.

## Discussion

Many studies have provided evidence of widespread HNRNPC binding to RNA [15, 17, 20, 32]. Indeed, HNRNPC binding to *MYC* RNA and the idea that HNRNPC can regulate *MYC* expression is not new [18]. Our contribution is independent validation of HNRNPC binding to *MYC* pre-mRNA in intron 2 with evidence of binding redundancy. The former was aided by the addition of the SF3B1 inhibitor pladienolide B to our RIP experiments. *MYC* intron 2 seemed to be short-lived, making it relatively rare in cells, but enrichment through splicing inhibition can enable detection of crucial interactions with introns like this that are constitutively spliced and that may otherwise be missed. Our latter contribution was demonstrated by the requirement that all three long poly(U) regions be mutated to disrupt HNRNPC binding. Correspondingly, reporter assays showed that mutation of all three poly(U) regions was required for modest but repeatable inhibition of *MYC* intron 2 splicing or translation. It is most likely that our reporter assays are predominantly assessing splicing fidelity; indeed, translational efficiency (protein activity per mRNA molecule) was not significantly different between the wild-type and mutant forms, though it trended that way (not shown). However, because we were unable to independently demonstrate intron retention of the three-region mutant versus the wild-type, we have been careful to note that some other effect on translation could be involved.

It is also important to note that the mutations in this study disrupted consecutive uridine residues but retained polypyrimidine stretches; polypyrimidine tract binding proteins necessary for splicing should not have been affected. However, our studies are unable to rule out the possibility that other poly(U)-specific binding proteins affect splicing of *MYC* intron 2 or translation. Neither direct binding nor loss of binding to the mutant reporter in the context of an intact cellular environment were examined. Thus, it is possible that the HNRNPC binding we observed was indirect and our mutant-altered reporter assay results are due to binding of other factors. Reasonable possibilities are FUBP1, TIA1 and/or TIAR; each of which have previously been identified as regulators of MYC expression [33–36]. FUBP1-mediated up-regulation of MYC was reportedly due to increased transcription, but subsequent studies that either reduced or eliminated FUBP1 from cells have seen no effect on the level of MYC [37, 38]. TIAR (*TIAL1* gene)–and possibly TIA1–regulation of MYC was through 3′UTR binding [35], but removal of TIA1 and TIAL1 genes in B cells did not alter MYC expression [39]. Comparatively, previous studies that have reduced HNRNPC expression have consistently shown reduced MYC expression [18, 40, 41]. Our mutagenesis studies are also consistent with the notion of multiple-site and simultaneous binding of HNRNPC–as both binding and reporter activity suggest a necessary redundancy for this association regarding regulation of constitutive splicing for this intron. The tetrameric form of HNRNPC is well established and current models indicate specific intervals for contact points on RNA [17, 32, 42]. Interestingly, there were two more poly(5U) regions in *MYC* intron 2 that we did not examine as they did not reach our initial eCLIP threshold score of at least 1000. Though there is increasing affinity of HNRNPC for longer poly(U) stretches [17], the two other poly(5U) regions, which happen to be the ones closest to the 5′ splice site and 3′ splice site, respectively, might be contributing to the splicing efficiency. Of note, the RNA used in our precipitation assays did not include either of those regions—and yet some HNRNPC binding remained. Overall, the combination of our finding that multiple intronic poly(U) regions can independently bind HNRNPC and mediate splicing or translational fidelity with the known multimeric association of HNRNPC to RNA suggests that this protein-pre-mRNA association is important. At the very least, it indicates that the poly(U) regions are important. That multiple poly(U) regions exhibit some level of conservation for intron 2 of *MYC* also lends support to this assertion.

The purpose behind examining intron 2 of *MYC* from different species was to determine whether poly(U) regions were a recurring theme for various *MYC* genes that had a similar architecture. Our species evaluation, while not exhaustive (overrepresentation of primates and two other human *MYC* genes), shows that these poly(U) regions do recur. For example, MAFFT and MUSCLE alignments showed 65% conservation for two poly(U) regions. Human *MYC* did have two spacing intervals close to 300 and one close to 150 nucleotides apart, which is consistent with those previously reported for human HNRNPC [17, 32], but overall, the exact distances between poly(U) regions were not extremely consistent across species. What was consistent is the proximity of these regions to one or the other splice junctions. The distance of the first poly(U) to the 3′ splice site and the distance of the last poly(U) to the 5′ splice site were remarkably correlated to the length of the intron across the species we examined (see S3 File Fisher’s Z tab). This fact lends support to the idea that these poly(U) regions in other species may also affect splicing or translation.

The presence of this interesting, correlated spacing indicated potential roles for RNA secondary structure, which has been suggested to alter the spacing and accessibility of splicing regulatory elements to modulate splicing [43]. This provided the impetus for analyzing *MYC* introns using ScanFold 2.0 [16] to identify regions with potential to be structured. Among old world primates, consistent secondary structures around what we call HNRNPC binding region 3 were revealed. Based on dimethyl sulfate (DMS) probing and modeling with a global refold analyses, an upstream hairpin was consistently present (Collin O’Leary, Taylor Eich, and Walter Moss unpublished). After modeling and mutagenesis in a reporter assay, it became clear that this small sequence also played a role in splicing inhibition. Our modeling suggests that the bulge enables flexibility to the terminal end of the hairpin, perhaps increasing the likelihood of suboptimal hairpin conformations that interfere with the splicing machinery. Another possibility is that the bulge region is directly involved in protein or RNA binding that slightly interferes with the splicing machinery. Many questions surrounding this remain to be explored, such as whether the observed de-repression is based on structure or sequence–folding or binding.

Indeed, there are many unresolved questions remaining from our study. How exactly does HNRNPC modify the splicing? What is the role of the other two poly(U) regions? What other factors are involved? Additionally, knowledge of HNRNPC binding to the *MYC* transcript is not new, but the idea that this could also be co-regulated by adenosine methylation (m^6^A) is a more recent and exciting development [12]. Both METTL3 and HNRNPC are known to regulate the level of *MYC* protein [18, 44, 45]. Liu et al. (2015) showed that a good portion of HNRNPC poly(U) binding regions are found close to METTL3 methylation sites and that methylation corresponds with greater binding [20]. Others have observed regulation of *MYC* abundance based on methylation in the 3′UTR [46], whereas in certain cells METTL3 knockdown does not change *MYC* protein levels [47]. Simply analyzing the sequence of intron 2, we found proximity between poly(U) regions and consensus m^6^A sites (S4 Fig). This potential interplay warrants further investigation.

Whether it’s shifting expression to an alternative start site [18], stabilizing the transcript through methylation [20], or potentially enhancing splicing (this work), the consistent theme is that HNRNPC promotes *MYC* expression. Better understanding exactly how HNRNPC binds and might modify *MYC* splicing may eventually provide a path to *MYC* inhibition.

## Supporting information

S1 Fig Denaturing gel showing biotinylated RNA product sizes after in vitro transcription. Biotinylated RNA was run on urea polyacrylamide gels (7% top; 5% bottom) after heating the RNA (0.5 μg) samples. Sample are as indicated (see Fig 3 for a diagram of the region that was transcribed). Wild-type (wt) was the originally cloned sequence, and the mutations (mut) introduced to each region and all combinations are as shown in Fig 3.
(TIF)

S2 Fig Poly(T) regions identified by MAFFT alignment of *MYC* introns. Two regions were identified that would be poly(U) regions after transcription. Alignments and image were generated using SnapGene 6.0 default conditions after input of *MYC* intron DNA fasta sequences as indicated. Consensus sequence threshold was set to 65%. Please see S3 File table values tab for species information.
(TIF)

S3 Fig Hairpin with asymmetrical bulges just upstream of poly(U) region in *MYC* intron 2 of old world primates. Poly(U) region for each sequence section is denoted by orange highlighting. Images generated using VARNA.
(TIF)

S4 Fig Secondary structure diagram of entire *MYC* intron 2 showing proximity of poly(U) and potential methylation sites. Poly(U) regions are identified in orange, and m^6^A consensus sequences are identified in blue. Regions that were expanded below are indicated by the black line. Secondary structure was predicted using ScanFold2.0 at a -1 z-score threshold and imaged using VARNA.
(TIF)

S1 File ScanFold2 output files for MYC intron 2 from selected species. (ZIP)

S2 File Extracted poly(5U+) regions for MYC intron 2 from selected species. (XLSX)

S3 File Poly(U) correlation coefficients and table values. (XLSX)

S4 File 3D movie of wild-type hairpin structure from Fig 5A. (MP4)

S5 File 3D movie of hairpin structure from Fig 5A with a six-nucleotide bulge deletion. (MP4)

S1 Raw images Raw images for blots represented in Figs 2D and 3B. (PDF)

## Abbreviations

eCLIP: enhanced cross-linking immunoprecipitation
HNRNPC: heterogenous nuclear ribonucleoproteins C1/C2
MYC: myelocytomatosis
RBP: RNA binding protein
RIP: RNA immunoprecipitation
