# Supplementary figures and images for: Identification of MYC intron 2 regions that modulate expression
Source: PLoS One. 2024 Jan 18;19(1):e0296889. doi: 10.1371/journal.pone.0296889 (PMC10795982; doi:10.1371/journal.pone.0296889)

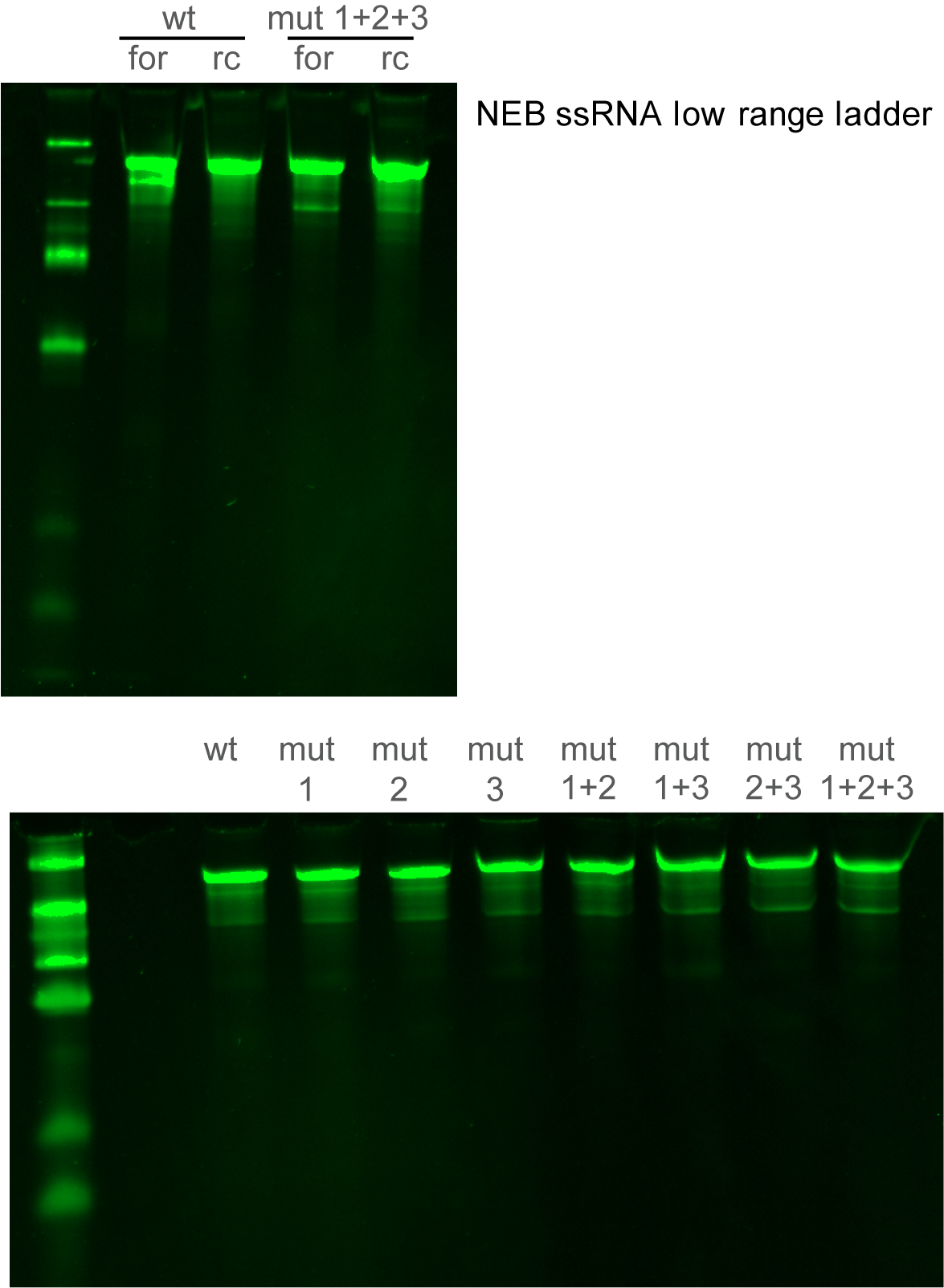

Supplement: S1 Fig — Biotinylated RNA was run on urea polyacrylamide gels (7% top; 5% bottom) after heating the RNA (0.5 μg) samples. Sample are as indicated (see Fig 3 for a diagram of the region that was transcribed). Wild-type (wt) was the originally cloned sequence, and the mutations (mut) introduced to each region and all combinations are as shown in Fig 3. (TIF) [file pone.0296889.s001.tif]

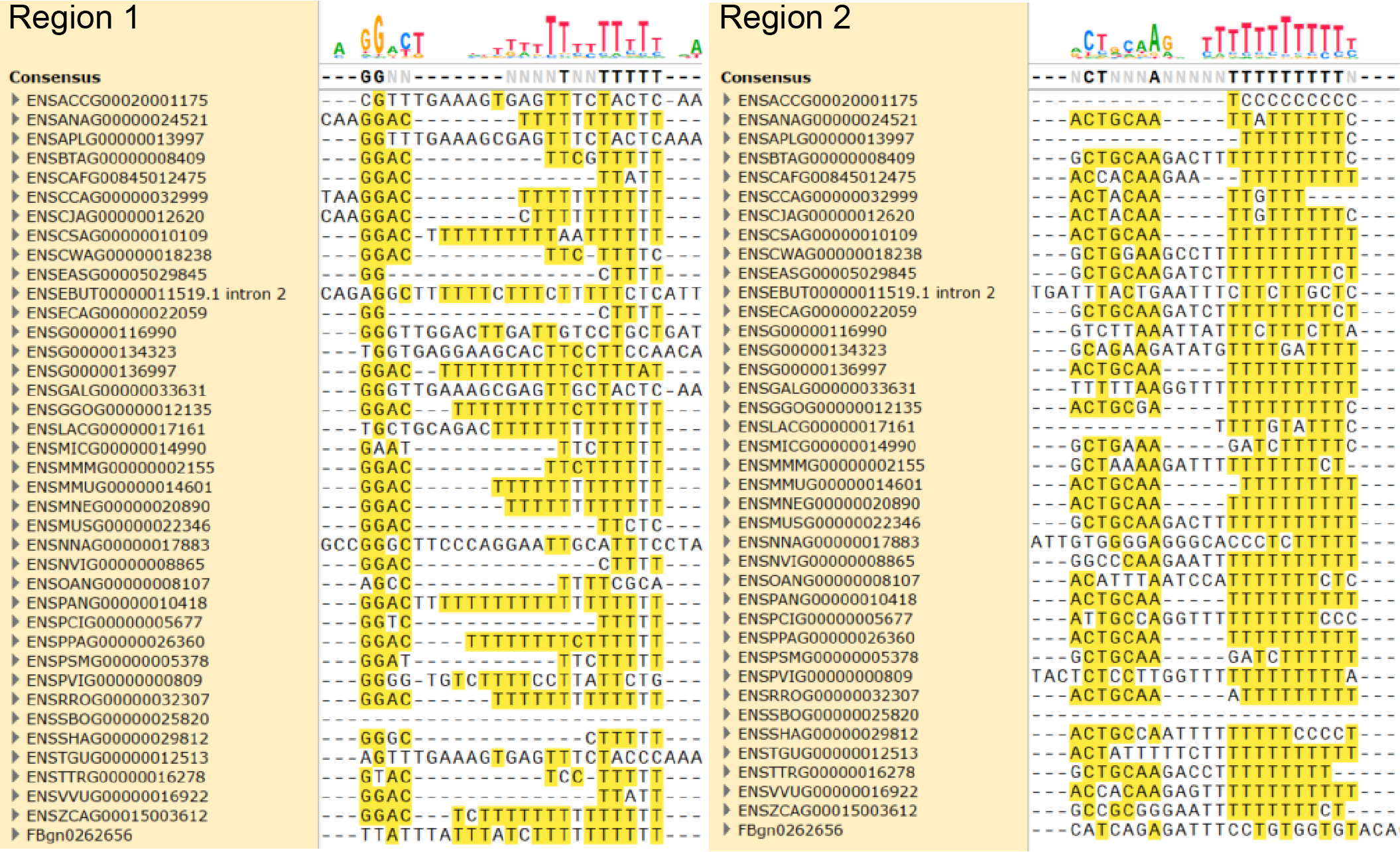

Supplement: S2 Fig — Two regions were identified that would be poly(U) regions after transcription. Alignments and image were generated using SnapGene 6.0 default conditions after input of MYC intron DNA fasta sequences as indicated. Consensus sequence threshold was set to 65%. Please see S3 File table values tab for species information. (TIF) [file pone.0296889.s002.tif]

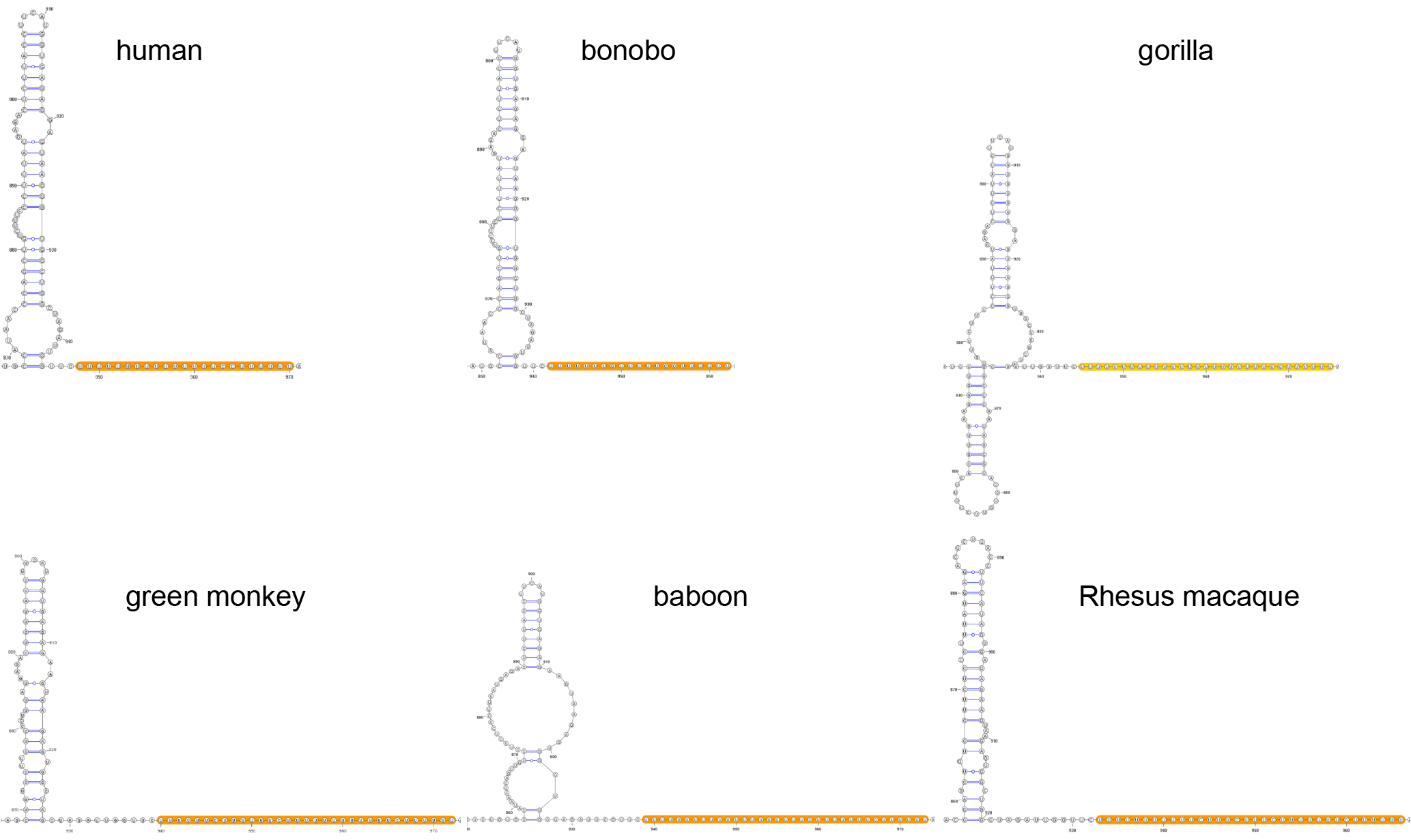

Supplement: S3 Fig — Poly(U) region for each sequence section is denoted by orange highlighting. Images generated using VARNA. (TIF) [file pone.0296889.s003.tif]

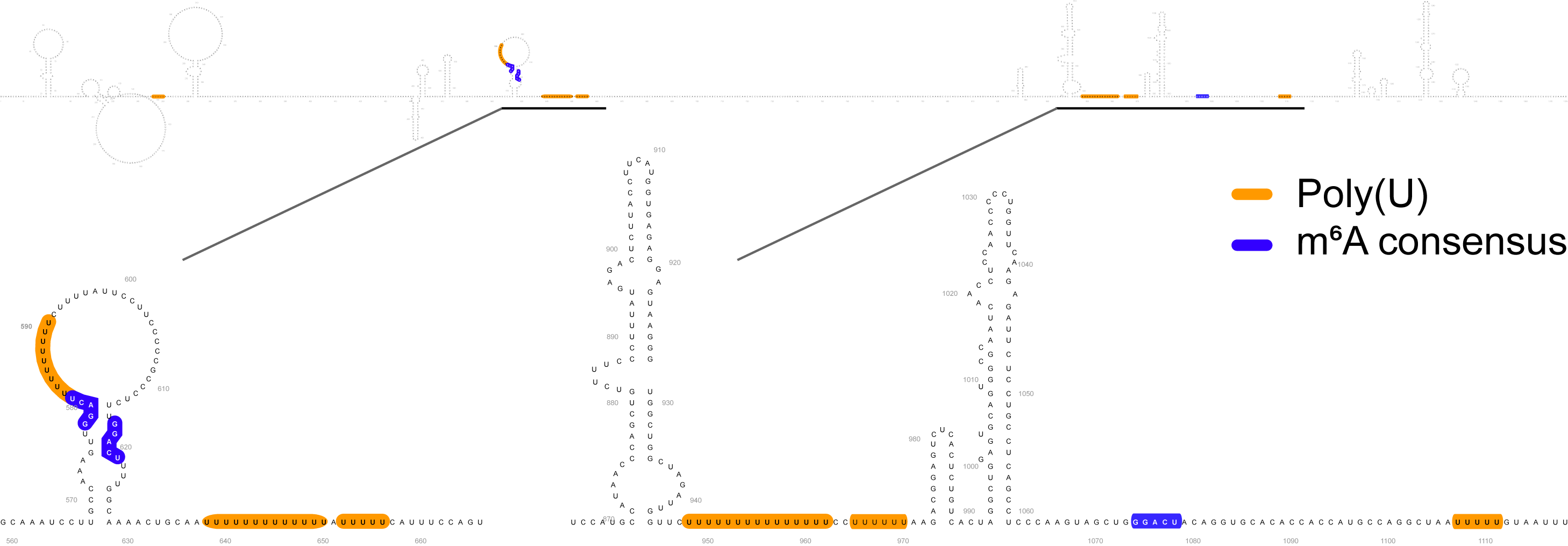

Supplement: S4 Fig — Poly(U) regions are identified in orange, and m6A consensus sequences are identified in blue. Regions that were expanded below are indicated by the black line. Secondary structure was predicted using ScanFold2.0 at a -1 z-score threshold and imaged using VARNA. (TIF) [file pone.0296889.s004.tif]
