# Supplementary material for: Identification of MYC intron 2 regions that modulate expression
Source: PLoS One. 2024 Jan 18;19(1):e0296889. doi: 10.1371/journal.pone.0296889 (PMC10795982; doi:10.1371/journal.pone.0296889)

From Figure 2D

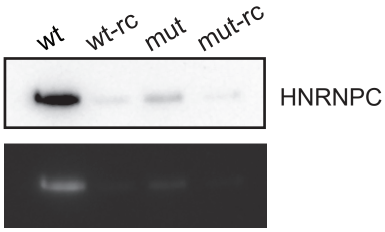

inverted and adjusted image

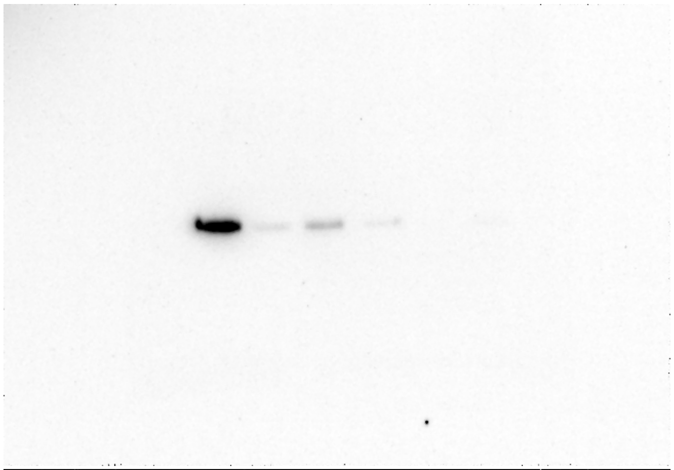

original image

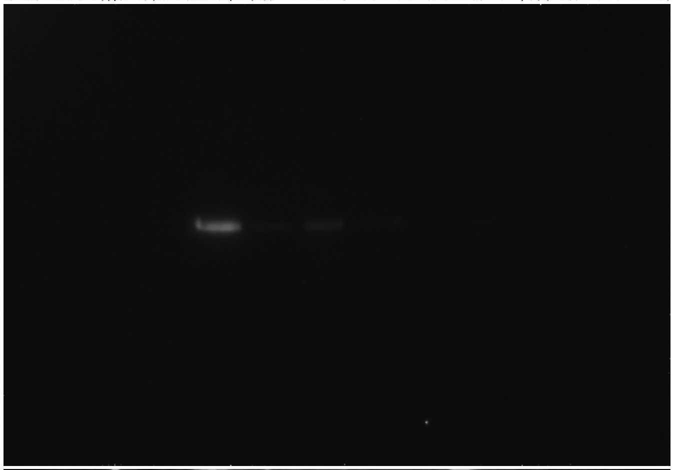

original image white light

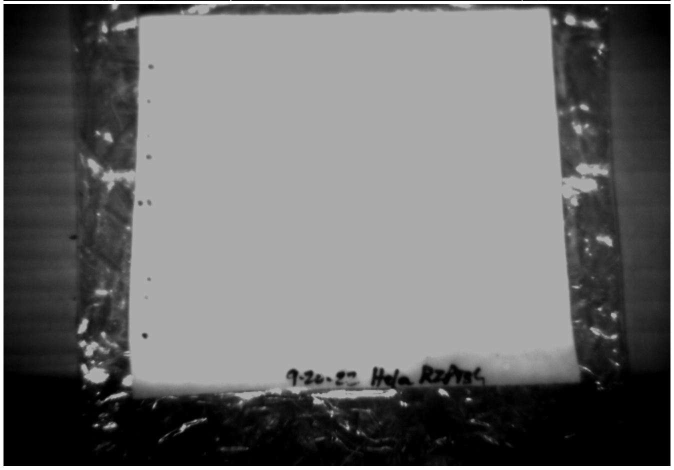

From Figure 3B

wt | mut 1 | mut 2 | mut 3 | mut 1+2 | mut 1+3 | mut 2+3 | mut 1+2+3

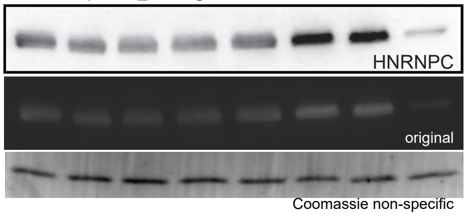

Repeat experiment with independently prepped lysate and bait RNA

10% Input | wt | mut 1+2+3 | mut 1 | mut 2 | mut 3 | mut 1+2 | mut 1+3 | mut 2+3

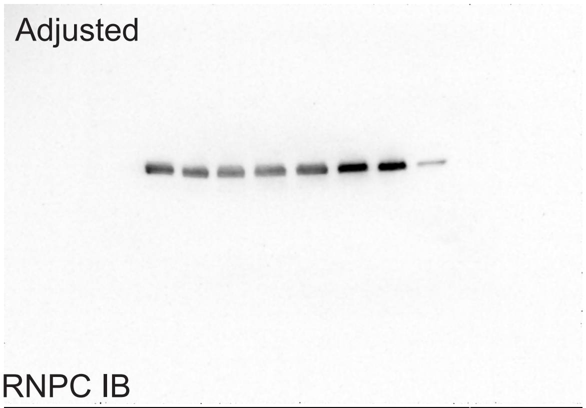

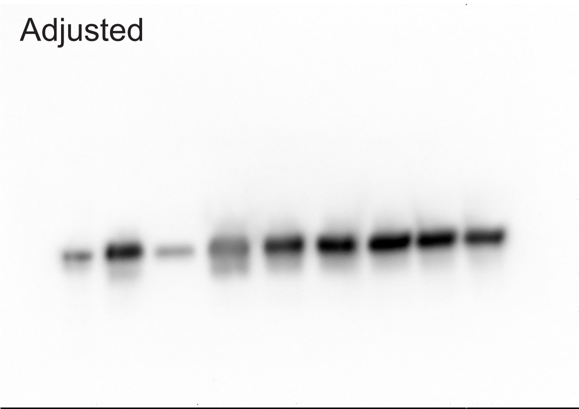

HNRNPC IB

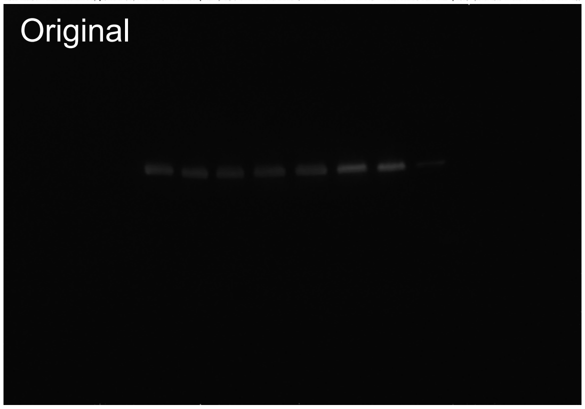

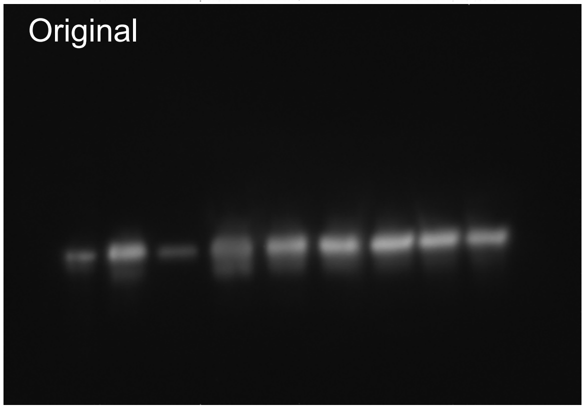

Adjusted

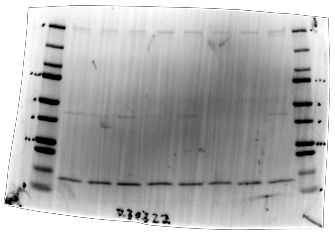

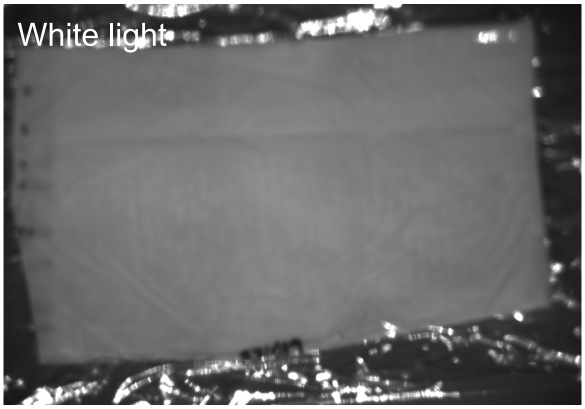

Coomassie stain

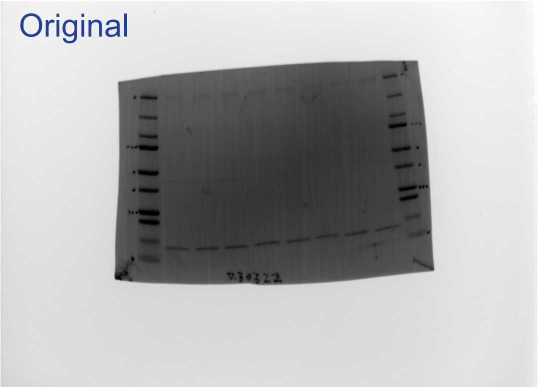

Supplement: S1 Raw images — (PDF) [file pone.0296889.s010.pdf]
